# Scurvy in a young man: a rare case report

**DOI:** 10.3389/fnut.2023.1265334

**Published:** 2023-10-17

**Authors:** Rui-Ling Lu, Jie-Wen Guo, Bao-dong Sun, Yu-Lan Chen, Dong-Zhou Liu

**Affiliations:** ^1^The Second Clinical Medical College, Jinan University (Shenzhen People’s Hospital), Shenzhen, China; ^2^Department of Rheumatology and Immunology, Shenzhen People’s Hospital, Shenzhen, China

**Keywords:** scurvy, vitamin C deficiency, dietary patterns change, staying indoors, lower extremities pain

## Abstract

Scurvy, resulting from vitamin C deficiency, has nonspecific constitutional symptoms, including weakness, malaise, and fatigue. It is frequently misdiagnosed due to the lack of specific clinical manifestations. Although there are sporadic cases of scurvy currently reported in children, scurvy in young people is seldom encountered. Here, we report on a 25-year-old male patient without any underlying conditions who presented with severe pain and ecchymoses of both lower extremities. He was diagnosed with scurvy due to a long history of staying indoors and inadequate intake of fruits or vegetables.

## Introduction

Scurvy, first reported in 1498, is a rare condition (1). Vitamin C deficiency in the diet, predominantly due to inadequate consumption of fresh fruit and vegetables, is the primary cause of scurvy (2). Nonspecific constitutional symptoms, including weakness, malaise, and fatigue, are common in scurvy (3). Skin hemorrhages, such as purpura and petechiae, are the most typical manifestations (3). Despite the occasional reports of scurvy in children (4–6), scurvy in young people without any underlying condition is uncommon. In the present case study, we describe a 25-year-old male patient without any underlying diseases who complained of severe pain and ecchymoses of both lower extremities and was diagnosed with scurvy during the coronavirus disease 2019 (COVID-19) pandemic.

## Case presentation

**Figure 1:**
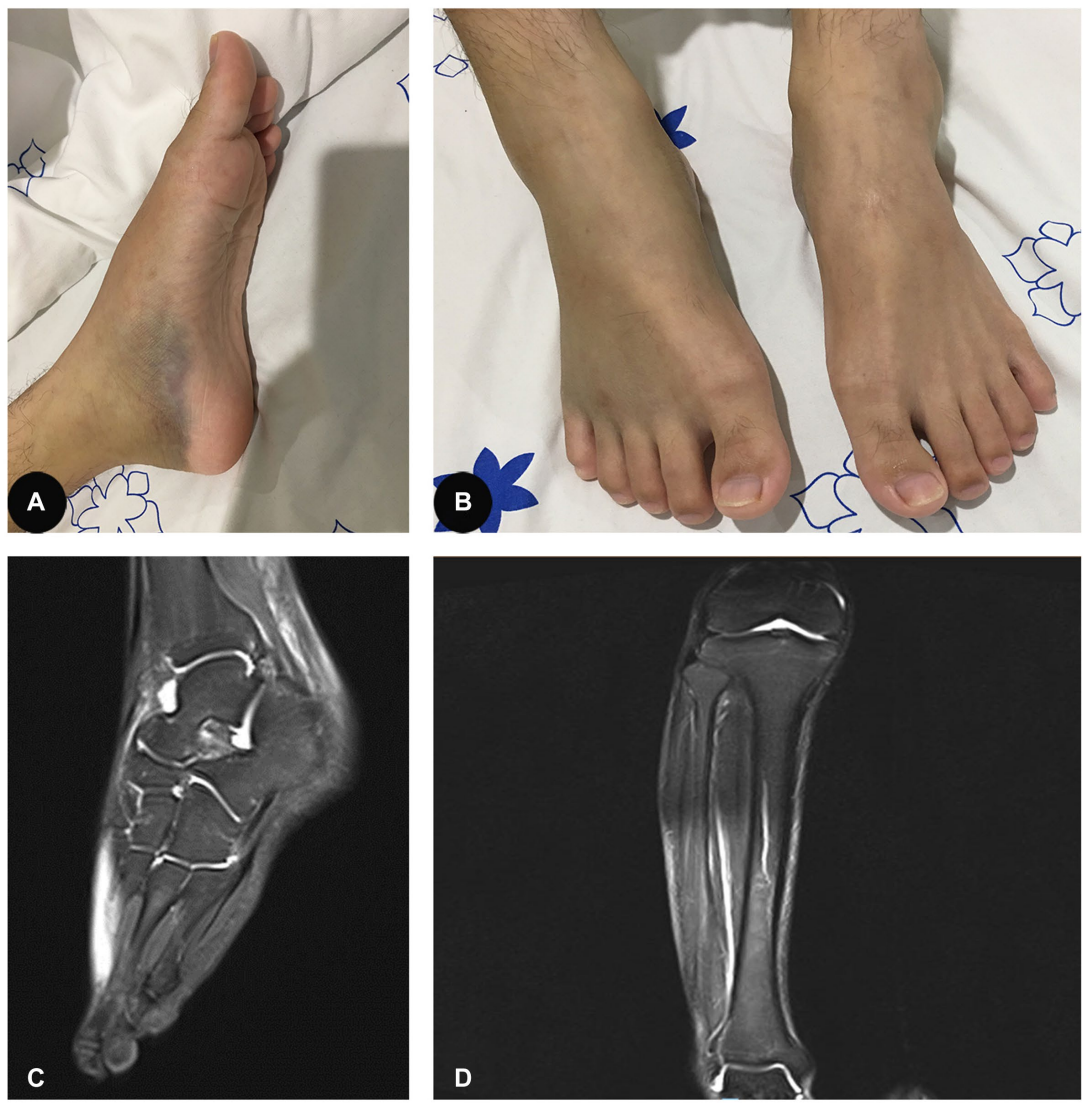
**(A,B)** Ecchymoses and mild swelling of both lower extremities. **(C)** Effusion in the right ankle capsule and swelling of the right posterior tibial tendon sheath, as well as the plantar, dorsal, and soft tissues around them. **(D)** Edema of the right calf interosseous muscle and effusion in the right knee.

A 25-year-old male patient was admitted to the hospital on August 3, 2020, due to a three-week history of progressively worsening pain and swelling of the bilateral lower extremities, with limited range of motion. He also complained of progressively worsening pain and swelling of his tongue, as well as significant weight loss (approximately 5 kg) over the previous 8 weeks. He did not complain of numbness of the extremities. His past medical history and family history was unremarkable. Physical examination on admission revealed a low body mass index of 17.34, swelling of the tongue and atrophy of the tongue papillae, ecchymoses and mild swelling of both lower extremities (Figures 1A,B), and tenderness in the right gastrocnemius muscle. There were no signs of muscle weakness or sensory abnormalities.

Blood tests suggested normocytic anaemia (hemoglobin 92 g/L, reference range: 120–160 g/L), a mild decline in leukocytes (3.78 × 10^9^/L, reference range: 4–10 × 10^9^/L), and an elevated level of D-dimer (2285.84 ng/mL, reference range: < 500 ng/mL). His basic metabolic panel, levels of complement components and the panel of autoantibodies were normal. C-reactive protein (CRP) was 4.84 mg/L (reference range: <5.0 mg/L) and erythrocyte sedimentation rate (ESR) was 9 mm/h (reference range: 0–15 mm/h). Coagulation function was normal, with prothrombin time of 13.3 s (reference range: 12 ± 3 s) and activated partial thromboplastin time of 34.9 s (reference range: 29.4 ± 10s). COVID-19 nucleic acid test was negative. Human leukocyte antigen-B27 was also within the normal range. The main laboratory data are shown in Table 1. Ultrasounds of both ankles and lower extremities revealed anterior recess effusion in the ankles. X-rays of both the lower extremities (including the long bones) and pelvis were unremarkable. There was no evidence to suggest malignancy or infection.

**Table 1:** Laboratory tests before and after treatment.

Parameter	Before	After	Reference range
Leukocytes (10^9^/L)	3.78	5.26	4–10
Erythrocytes (10^12^/L)	3.24	3.89	4–5.5
Hemoglobin (g/L)	92	137	120–160
Platelets (10^9^/L)	292	267	100–300
Erythrocyte sedimentation rate (mm/h)	9	3	0–15
C-reactive protein (mg/L)	4.84	ND	<5
PT (s)	13.3	ND	12 ± 3
APTT (s)	34.9	ND	29.4 ± 10
Fibrinogen (g/L)	3.39	ND	2–4
D-dimer (ng/ml)	2285.84	ND	68–494

PT, prothrombin time; APTT, activated partial thromboplastin time; ND, not detected.

Although nonsteroidal anti-inflammatory drugs were administered, no improvement was observed in the patient. Magnetic resonance imaging (MRI) further suggested oedema of the right calf interosseous muscle and a slight effusion in the right knee (Figure 1C). In addition, there was a slight effusion in the right ankle capsule and swelling of the right posterior tibial tendon sheath, as well as the plantar, dorsal, and soft tissues around them (Figure 1D). Malnourished anaemia was suspected due to low levels of folate (6.68 nmol/L, reference range 10.4–42.4 nmol/L) and vitamin B12 (72 pmol/L, reference range 350–1,200 pmol/L). A more detailed history revealed a fast-food diet and an extreme lack of fruit and vegetable consumption over the last several months during the peak of the COVID-19 pandemic. Therefore, scurvy due to malnutrition was highly suspected. However, the patient refused to have the concentration of plasma vitamin C measured further because it was not available in our hospital and his health insurance did not cover the medical expense. Therefore, oral vitamin C (0.2 g three times daily), folate (10 mg three times daily), and mecobalamin supplementation (0.5 mg three times daily) were immediately initiated for the patient. Intriguingly, pain and swelling of the bilateral lower extremities and tongue improved gradually. Four weeks later, his original symptoms were almost completely resolved, with normal levels of leukocytes, hemoglobin, folate and vitamin B12, and he come back to a normal life with satisfaction of the outcome of our treatment. Three months later, the patient stopped those supplementations by himself. He was followed up with a phone call over the next year and he did not experience any recurrence of the original symptoms.

## Discussion

Scurvy was first reported in 1498, and there were several outbreaks of scurvy among sailors in the 16th and 17th centuries. However, it was not until the 20th century that people realized scurvy was caused by vitamin C deficiency (1). Although scurvy is uncommon today, a few cases have been reported in recent years, most of which are in elderly individuals, children, or people in lower socioeconomic areas (2, 4–8). Scurvy in young people has been primarily found in patients with underlying conditions, such as anorexia nervosa, psychiatric disorders, and specific dietary restrictions (9–11). Cases in young people with unremarkable past medical histories are extremely uncommon (12). To the best of our knowledge, only six cases of young scurvy patients (18–40 years) without any underlying conditions have been previously reported (13–18) (Table 2).

**Table 2:** Young scurvy patients with unremarkable past medical histories previously reported.

Author, year	Region	Age	Gender	Clinical manifestations	Treatment	Outcome
Davies, 1967	Africa	27	M	Leg pain, gingival bleeding, petechiae	Vitamin C 700 mg/d	Recovery
Shelton, 1992	America	20	F	Ecchymosis, petechiae	Vitamin C 500 mg/d	Recovery
Firth, 2001	Oceania	19	M	Gingival bleeding, hemoptysis	Increased intake of vitamin C in the diet	Lost to follow-up
Koçak, 2003	Türkiye	32	M	Leg pain, ecchymosis	Vitamin C 1–2 g/d (for the first 2 days), followed by 500 mg /d	Recovery
Mertens, 2011	America	26	M	Leg pain, purpuric	Vitamin C 1 g/d	Recovery
Pallant, 2022	America	26	M	Muscle soreness, ecchymosis, petechiae, gingival bleeding	Vitamin C 1 g/d	Recovery

Scurvy results from vitamin C deficiency, which is a crucial cofactor for collagen enzymes. Vitamin C is necessary in the process of hydroxylation catalyzed by prolyl-4-hydroxylase and lysyl hydroxylases (19). Prolyl residues must be hydroxylated to form stable triple-helical collagen, while lysyl must be hydroxylated to initiate collagen crosslinking (19, 20). According to the Chinese Dietary Reference Intakes, the estimated average need for vitamin C in adults is 85 mg per day (21). However, the level of plasma vitamin C at which scurvy develops is still not definitive. Scurvy is reported to occur when the total vitamin C content in the body decreases to less than 300 mg and the levels in the plasma are below 10 μM (22).

Scurvy is characterized by nonspecific constitutional symptoms such as weakness, malaise, and fatigue (3). Skin hemorrhages are the most common symptom, which may be attributed to defective collagen synthesis caused by vitamin C deficiency. Folic hyperkeratosis and corkscrew hairs, which are the classic skin findings of scurvy, were present in almost all previously reported cases of adult patients with scurvy (23). Furthermore, it has been estimated that approximately 80% of patients with scurvy suffer from musculoskeletal symptoms, such as arthralgia, myalgia, and muscular haematomas (24). Patients can feel tired if the levels of plasma vitamin C are below 20 μM (19). This may be caused by a reduction in carnitine synthesis, which is essential in the process of fatty acid oxidation in tissues, especially muscles (19). In addition, hemorrhage into the muscles and other soft tissues might also partially explain the discomfort (1). Accordingly, ecchymoses on both lower extremities as well as significant myalgia and arthralgia also occurred in our patient. Notably, tongue pain was evident in our patient, which has never been reported previously in patients with scurvy. Nevertheless, patients with anaemia may suffer from tongue pain due to atrophic glossitis (25).

Laboratory findings in patients with scurvy are unspecific. Normocytic anaemia with an increase in reticulocytes can be present due to a high incidence of bleeding in patients with scurvy (17). Folic acid deficiency and iron malnutrition might be concurrent contributory causes of anaemia (3). Folic acid is essential for ingesting intestinal iron and is primarily found in fruits and vegetables that contain vitamin C (3). Anaemia may also result from intravascular haemolysis (3). Most (4/6) young scurvy patients with unremarkable past medical histories demonstrate mild anaemia. Two of these patients had a mildly elevated bilirubin, which may be caused by haemolysis (1), while the other two patients had a slightly elevated erythrocyte sedimentation rate. This may be attributed to increased levels of inflammatory chemokines stimulated by vitamin C deficiency (3). An elevated D-dimer level can be associated with acute phase response. However, in this case, the classic indicators of acute phase response, CRP and ESR, were within normal ranges. Therefore, the elevated level of D-dimer in this patient may also be related to anaemia (26). Importantly, a low concentration of plasma vitamin C is often direct evidence of scurvy, while a rapid clinical response to vitamin C supplementation allows for confirmation of the diagnosis without laboratory studies (3). In the present case study, there were no specific laboratory findings except for orthochromatic anaemia, mild leukopenia, and elevated D-dimer in the patient, leading to difficulty in the diagnosis of scurvy. A lack of plasma vitamin C in this patient is a limitation of the case report. This patient was admitted to our hospital mainly due to progressively worsening pain and swelling of the bilateral lower extremities. It seems to be difficult to completely explain it by a lack of folate and vitamin B12, which mainly causes anemia and neurological symptoms, such as numbness and paresthesia of the extremities. However, he did not have any complaints of numbness, and physical examination on the admission did not find any sensory abnormalities. Therefore, the diagnosis of scurvy was made mainly based on the clinical data and his rapid clinical response to treatment in this patient.

Dietary patterns changed during the peak of the COVID-19 pandemic. Charles et al. reported on the first case of a 28-year-old male patient who suffered from scurvy during the COVID-19 pandemic due to a long stay-at-home history without intake of fruits or vegetables (27). He had a medical history of major depression, anxiety, and attention deficit hyperactivity disorder. He also reported intermittent marijuana use and an increase in alcohol consumption during the pandemic, which may also contribute to the occurrence of scurvy. Here, we report on the first case without any underlying diseases or substance abuse during the peak of the COVID-19 pandemic. Since scurvy is extremely rare, especially in young patients, it can be challenging to make a definite diagnosis. Therefore, clinicians should not ignore the possible diagnosis of scurvy in high-risk patients.

## Conclusion

Scurvy is currently a rare disease, and it is particularly difficult to make a definite diagnosis due to a lack of specific clinical manifestations. Early diagnosis and timely treatment are pivotal to avoid life-threatening consequences in the advanced stage of scurvy. Therefore, clinicians should be aware of the diagnosis of scurvy in high-risk populations, especially during challenging times such as pandemics.

## Data availability statement

The original contributions presented in the study are included in the article/supplementary material, further inquiries can be directed to the corresponding authors.

## Ethics statement

Ethical approval was not required for the studies involving humans because this was a retrospective descriptive study and patient consent was obtained for publication. The studies were conducted in accordance with the local legislation and institutional requirements. The participants provided their written informed consent to participate in this study. Written informed consent was obtained from the individual(s) for the publication of any potentially identifiable images or data included in this article. Written informed consent was obtained from the patient for the publication of this case report.

## Author contributions

R-LL: Conceptualization, Data curation, Investigation, Methodology, Writing – original draft. J-WG: Conceptualization, Data curation, Methodology, Project administration, Writing – original draft. B-dS: Taking photos, Following up the patient, Writing – review & editing. Y-LC: Formal analysis, Investigation, Methodology, Supervision, Validation, Visualization, Writing – review & editing. D-ZL: Funding acquisition, Resources, Supervision, Validation, Visualization, Writing – review & editing.
